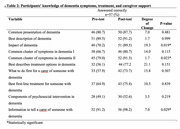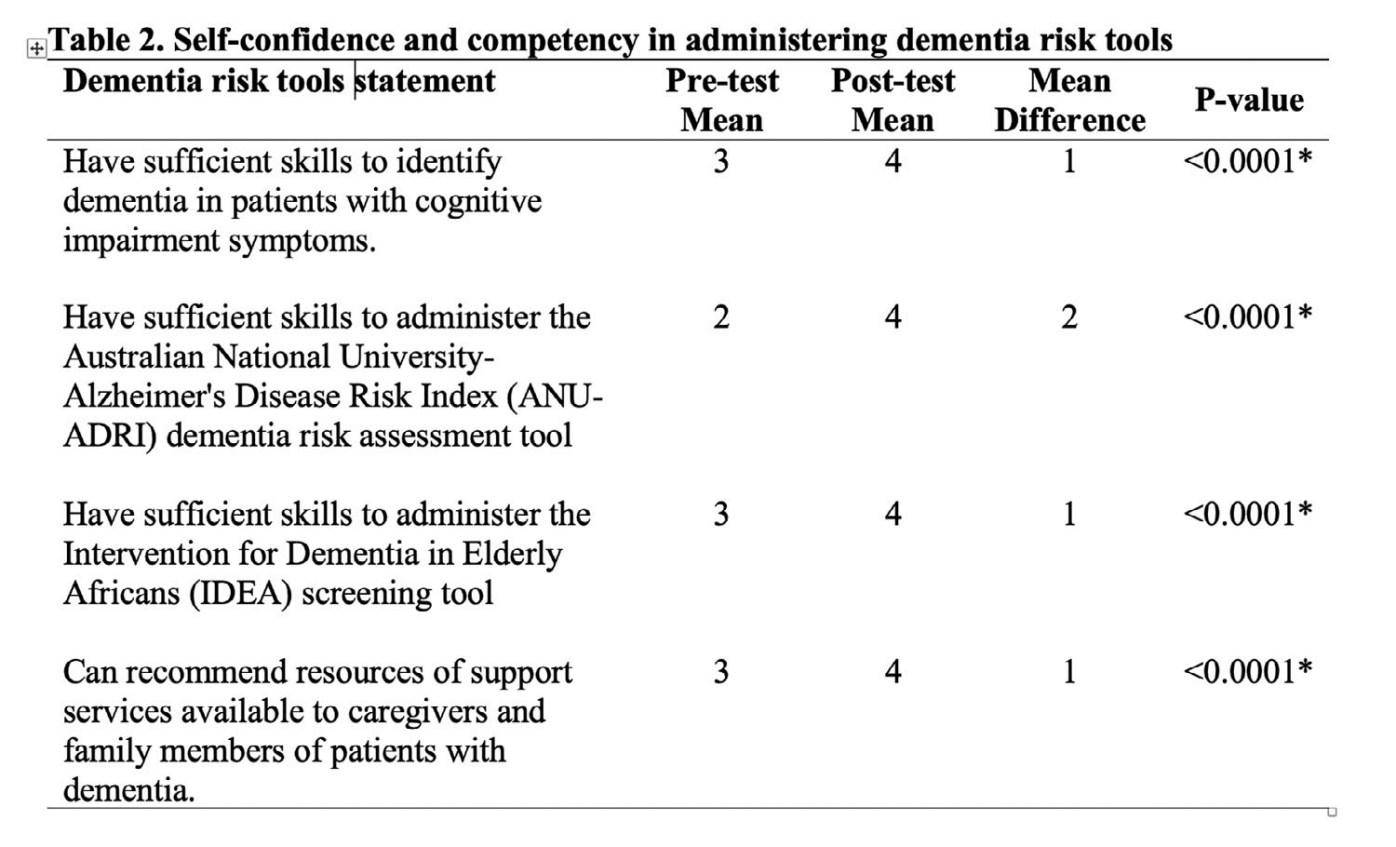# Integrating Dementia Risk Reduction into Primary Healthcare: Evaluating a Training Program for Health Educators in Nigeria

**DOI:** 10.1002/alz70858_099931

**Published:** 2025-12-25

**Authors:** Adedoyin O Ogunyemi, Adedunni W Olusanya, Njideka U Okubadejo, Lea T. Grinberg

**Affiliations:** ^1^ University of Lagos, Lagos, Lagos, Nigeria; ^2^ Global Brain Health Institute, University of California San Francisco, San Francisco, CA, USA; ^3^ College of Medicine, University of Lagos, Lagos, Nigeria; ^4^ Memory and Aging Center, UCSF Weill Institute for Neurosciences, University of California, San Francisco, San Francisco, CA, USA; ^5^ Global Brain Health Institute, University of California, San Francisco, San Francisco, CA, USA

## Abstract

**Background:**

The burden of dementia is high in low‐ and middle‐income countries, warranting interventions for primordial prevention. Integration of risk reduction strategies into primary health care can be achieved by leveraging existing manpower such as health educators, and providing training to deliver evidence‐based dementia prevention strategies to communities.

**Method:**

This study utilized a mixed methods design, and was conducted in Lagos State, southwestern Nigeria. One health educator was selected and trained in each of the 57 government districts. The training curriculum developed for the project included dementia recognition, risk reduction, and practical fieldwork using the WHO mhGap training manual (Dementia module), the ANU‐ADRI tool, and the IDEA cognitive screening tool. Quantitative pre‐ and post‐training assessments evaluated participants’ knowledge, skills, and confidence, while focus group discussions explored the feasibility, capability, and acceptability of the intervention.

**Result:**

The training improved participants' knowledge of dementia symptoms, treatment options, and caregiver support, with notable increases in correct responses, such as knowledge of the impact of dementia. (70.2% to 89.5%, *p* = 0.02). Attitudes toward dementia care also shifted positively, with significant changes in perceptions of treatment importance and the role of primary healthcare teams. Participants demonstrated enhanced confidence and competency in using dementia risk assessment tools, including the ANU‐ADRI and IDEA tools (*p* < 0.0001). Improvements in knowledge of dementia risk reduction strategies were observed, specifically in lifestyle‐related items (*p* = 0.02).

**Conclusion:**

The training program effectively improved participants' knowledge, attitudes, and competency in dementia recognition, perceptions, and risk reduction, highlighting its potential for integration into primary healthcare systems.